# Preserving Creativity in Medicine

**DOI:** 10.1371/journal.pmed.0010034

**Published:** 2004-12-28

**Authors:** David A Shaywitz, Dennis A Ausiello

## Abstract

Imagination and creativity are essential traits that medicine, and medical insurers, must again learn to recognize and reward

Galvanized by rising costs, increased calls for greater, accountability, and an Institute of Medicine (Washington, D. C., United States) report suggesting that medical errors may kill nearly 100,000 Americans every year [1], United States health care experts have tried to boost the quality of patient care by focusing on the speed and precision of service delivery. Several insurance companies have already started to place a surcharge on patients who elect to receive care from “inefficient” providers (a definition that includes most teaching hospitals), hoping to encourage patients to seek more cost-effective service, and to encourage physicians to provide it [2].

The problem is, most of these reform efforts, while critically important, only capture half the picture. Efficiency isn't everything, and unless we learn to cultivate creativity as avidly as we pursue consistency, future generations of patients may find themselves stuck with the same basic treatments they're receiving today. It will be the same medicine, just served quickly.

## Benefits of Quality Reform

From its earliest days, medical training was based on an apprenticeship model, in which junior acolytes learned the art from senior practitioners. Even with the evolution of modern medical schools, which offered future physicians a rigorous common training, once doctors entered the real world they essentially did as they pleased. Consequently, there were pronounced differences in approaches to common problems from one clinician to another.

There was also little to guarantee that once doctors had hung out their shingle, they were actually competent (and remained competent) to practice their craft. While most physicians remained committed to the general professional standard—do the best that you can for each individual patient— many well-meaning doctors ultimately were not delivering their patients the best care available.

More recently, and largely due to the contagious spread of the so-called “business model,” there has been an increased emphasis on the consistency and quality of care. The clear goal is ensuring that all patients truly receive the very best care available, as defined by rigorous scientific studies.[Fig pmed-0010034-g001]

**Figure pmed-0010034-g001:**
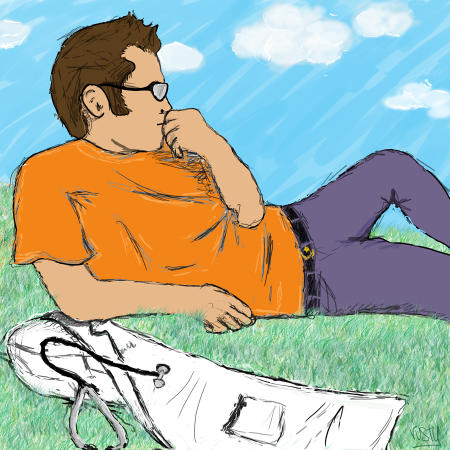
Contemplation can provide new medical insights (Illustration: Rusty Howson, sososo design)

This discrepancy between what patients should be receiving and what patients are actually receiving is the major focus of quality reform, and reflects the new recognition that there are truly preferred approaches—pathways—to guide disease management. These pathways are not meant to represent a rigid algorithm reflexively applied to each patient, but are intended as a summary of the best available data, a useful template to guide further medical decisions.

The renewed emphasis on quality has also resulted in a newfound appreciation for the role of experience and repetition in patient care. Study after study has shown that the best physician to treat a particular problem is the one who has treated it the most [3].

## What Gets Lost: Innovation

The great paradox here is that the same reforms that are improving our current care may also be endangering our future health. As medicine has become more standardized and increasingly regulated, it turns out there is much less room for innovation. The spirited pursuit of the unknown—so long a defining quality of medicine—now seems seriously endangered. The new world of rapid throughput and endless documentation provides little time to reflect upon important clinical problems and consider fresh approaches. If anything, thinking about a patient or a question too much is now implicitly discouraged because it slows doctors down; contemplation is bad for productivity.

Academic medical centers like our own have played a particularly important role in the history of medical discovery; the hallmark of these institutions is our commitment to thinking and reflecting about the patients we see, patients who are often extremely sick and whose management is exceptionally complex. Unfortunately, many of the measurements now used by insurance companies to assess quality pay little attention—if any at all—to the complexity of a patient's illness, or to the importance of spending time trying to define the underlying malady. Insurance companies' major concern seems to be how fast a patient is “processed,” ideally with as few tests as possible. These measures provide no mechanism for distinguishing between the addled physician who inappropriately orders every test that springs to mind, and the reflective physician who is trying to get to the bottom of a patient's complaint, rather than simply throw a Band-Aid over the symptoms [4].

Situated on the front lines, clinicians have a unique opportunity to provide new medical insights and to identify critical, unanswered questions. Classic examples include Archibald Garrod, a British physician whose desire to understand why a patient produced black urine led to the hypothesis that diseases can result from defective metabolic enzymes, and Fuller Albright, a clinical investigator at Harvard whose thoughtful approach to his patients yielded insights that revolutionized the field of endocrinology. More recently, the astute clinical observations of UCLA immunologist Michael Gottlieb resulted in the original description of the Acquired Immune Deficiency Syndrome (AIDS) in 1981 [5].

## Preserving Creativity in Medicine

But where are these types of insights going to come from today? It seems difficult to imagine that a medical care environment characterized by staccato-quick patient visits covering an ever-increasing number of compulsory topics will support or encourage such reflection and innovation. Our failure to nourish and sustain inquisitive physicians seems particularly tragic because medicine has traditionally attracted some of our brightest and most imaginative individuals. Even at the height of the dot-com boom, for example, there were still more medical school applicants than there were spaces to train them. But if current trends continue, many of these creative minds will head elsewhere, while those who stay will risk becoming stultified by repetitious routine.

Several medical schools and a handful of foundations have recognized this emerging problem, and have initiated programs aimed at sparking curiosity in young doctors (our own school's program is called the PASTEUR initiative—see www.pasteur.hms.harvard.edu) [6]. But as well-intentioned as these efforts are, simply changing the curriculum isn't likely to fix the underlying problem. Unless ever-savvy medical students perceive that inquisitive thinking is truly valued in clinical medicine, and unless exasperated physicians are inspired to believe that they have the ability to change some aspect of the way medicine is practiced, nothing is going to change. We may lose the best hope we have of defeating the terrible diseases that now plague us.

Even as we strive to improve the consistency of care—and striving is clearly a very good idea—we must continue to cultivate novelty and originality, rather than penalize it. Imagination is perhaps the most essential trait that medicine, and medical insurers, must again learn to recognize and reward. Even with the best algorithms and the brightest computers, the future of health care ultimately depends upon the creativity of the hardy men and women still entrusted with its delivery.
